# Macrophage-derived exosomes mediate glomerular endothelial cell dysfunction in sepsis-associated acute kidney injury

**DOI:** 10.1186/s13578-023-00990-z

**Published:** 2023-03-07

**Authors:** Huiling Xiang, Zhifeng Xu, Chun Zhang, Jing Xiong

**Affiliations:** Department of Nephrology, Union Hospital, Tongji Medical College, Huazhong University of Science and Technology, Wuhan, China

**Keywords:** Exosome, Macrophage, Sepsis, Acute kidney injury, Acid sphingomyelinase

## Abstract

**Background:**

Sepsis-associated AKI has been shown to be related to sepsis mortality. Macrophage activation and endothelial cell damage are involved in the progression of sepsis-associated AKI, but the specific mechanisms are still unclear.

**Methods:**

In vitro experiments, exosomes extracted from lipopolysaccharide (LPS) -stimulated macrophages were co-incubated with rat glomerular endothelial cells (RGECs) and then detected the injury markers of RGECs. Acid sphingomyelinase (ASM) inhibitor amitriptyline were used to investigate the role of ASM. In vivo experiment, exosomes produced by LPS-stimulated macrophages were injected into mice through tail vein to further explore the role of macrophage-derived exosomes. Moreover, ASM knockout mice were used to verify the mechanism.

**Result:**

In vitro, the secretion of macrophage exosomes increased upon the stimulation with LPS. Notably, macrophage-derived exosomes can cause glomerular endothelial cell dysfunction. In vivo, macrophage infiltration and exosome secretion in glomeruli of the LPS-induced AKI group increased. The exosomes produced by LPS-stimulated macrophages were injected into mice, which also led to the injury of renal endothelial cells. In addition, in the LPS-induced AKI mouse model, compared with wild-type mice, the secretion of exosomes in glomeruli of ASM gene knockout mice and the injury of endothelial cells were reduced.

**Conclusion:**

Our study shows that ASM regulates the secretion of macrophage exosomes, leading to endothelial cell injury, which may be a therapeutic target in sepsis-associated AKI.

**Supplementary Information:**

The online version contains supplementary material available at 10.1186/s13578-023-00990-z.

## Introduction

Sepsis-associated acute kidney injury (AKI) is an acute organ dysfunction syndrome, which is the most common complication in the intensive care unit and accounts for approximately 50% of all types of AKI [1]. Although the treatment of sepsis-associated AKI has been greatly improved in the past, the mortality rate remains high [2]. The main pathological manifestations of sepsis-associated AKI are renal tubular injury, endothelial cell injury, and immune cell infiltration [3]. Endothelial cell dysfunction is mainly manifested in the transformation of healthy endothelial cells into damaged procoagulant and pro-inflammatory phenotypes [4]. Immune cells participate in the process of inflammation and injury repair in the early stage of AKI and communicate closely with endothelial cells. However, the specific mechanism is still unclear [5].

Exosomes (EXOs) are extracellular vesicles (EVs) that are important for information exchange between cells [6]. EXOs is mainly produced by multivesicular bodies (MVBs), with a diameter of 30–100 nm, and contain proteins, lipids, RNA, etc. [7]. EXO-associated information exchange may be positive or negative, depending on the physiological conditions [8]. For example, in a fibrotic environment, EXOs produced by glomerular epithelial cells play a role in promoting fibrosis; while in a hypoxic environment, EXOs produced by renal tubular epithelial cells have a protective effect on AKI [9, 10].

Previous studies have confirmed that EXOs secreted by immune cells, especially macrophages, are involved in intercellular communication [11–13]. For example, EXOs produced by renal tubular cells stimulated by albumin can activate macrophages, and EXOs produced by macrophages play a role in renal calcification [7, 8]. However, it is unclear whether there is information exchange between EXOs produced by infiltrating macrophage cells and glomerular endothelial cells in the glomeruli of sepsis-associated AKI. Acid sphingomyelinase (ASM), which is mainly expressed on the surface of lysosomes, is an important lysosomal hydrolase. It hydrolyzes sphingomyelin to ceramide, which forms a curved enrichment platform on the cell membrane surface to facilitate EXO transport [14, 15]. Recent studies have shown that ASM also mediates the endocytosis process and participates in the formation of early endosomes [14].

Therefore, the purpose of this study was to investigate the role of macrophage-derived EXOs in lipopolysaccharide (LPS)-induced AKI glomerular endothelial cell injury and whether ASM is involved in the regulation of EXO secretion.

## Result

### Macrophage release more EXOs under LPS stimulation than under normal conditions

**Fig. 1:**
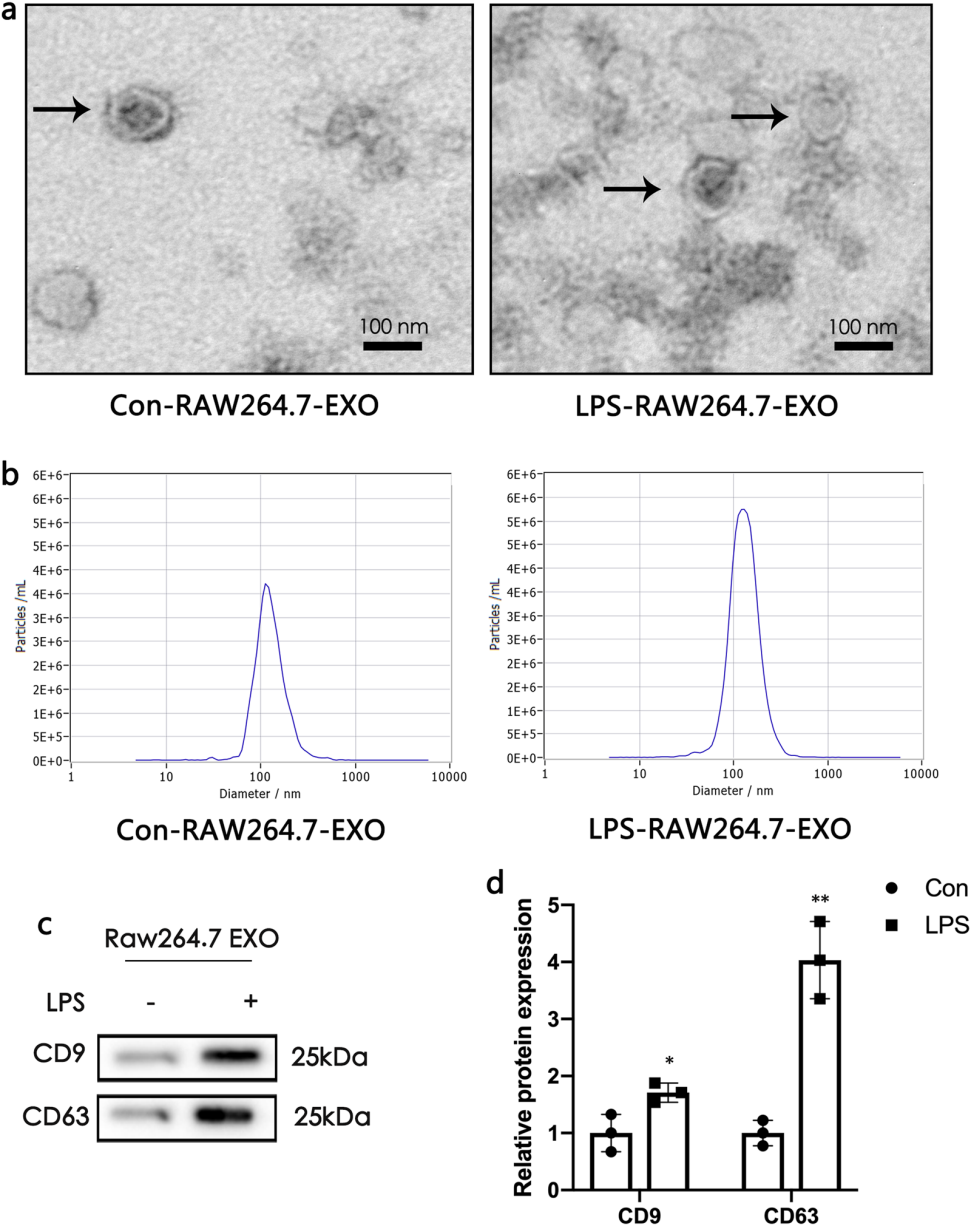
Macrophage produce more exosomes (EXOs) under lipopolysaccharide (LPS) stimulation. **a** Morphology of RAW264.7-EXO observed by electron microscopy. The scale bars represent 100 nm. **b** Measurement of RAW264.7-EXO population by nanoparticle tracking analysis (NTA) demonstrated a single-peaked pattern. **c **and** d** Expression of EXO markers [cluster of differentiation (CD)9 and CD63] was detected by western blotting. Data represent the mean ± SEM in independent experiments. ^*^P < 0.05, ^**^P < 0.01 versus Con

As shown in Fig. 1a, the typical double-layered membrane structure of EXOs with a diameter of approximately 100 nm can be observed under a microscope. Most of the extracted EXOs have a similar particle size distribution and are single-peak, indicating that the sample has a high purity (Fig. 1b). The expression of EXOs markers CD9 and CD63 in the LPS groups was higher than in the control group. This result shows that LPS stimulation increases the EXO secretion by macrophages (Fig. 1c, d).

### Macrophages infiltration and EXO release are increased in an LPS-induced AKI model

**Fig. 2:**
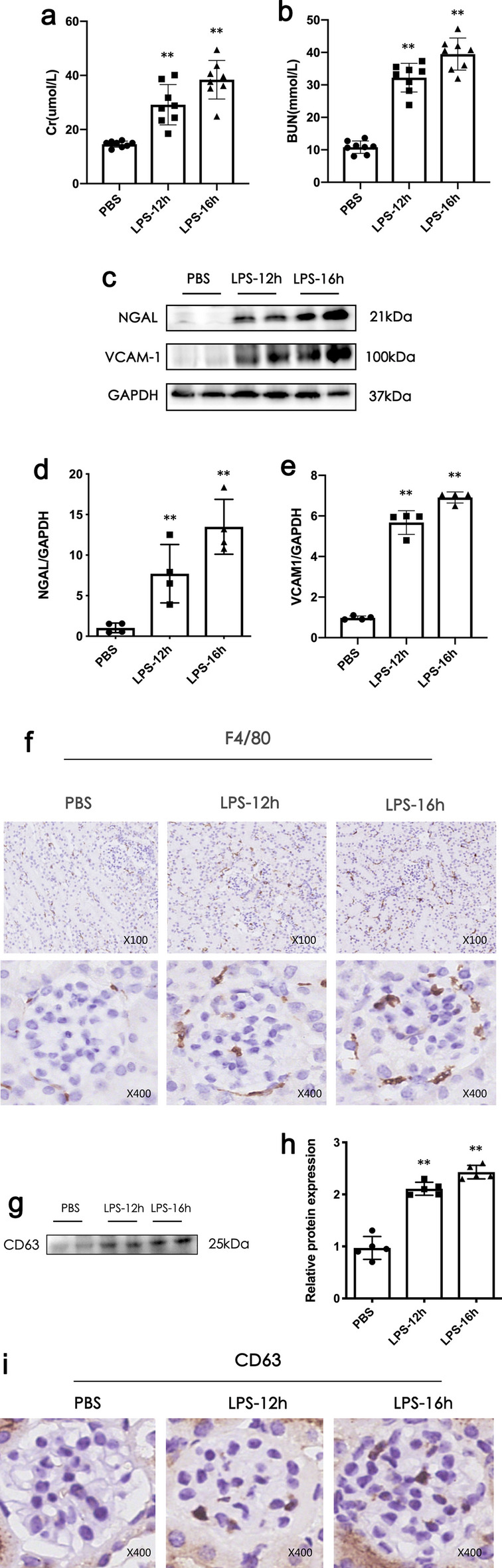
Macrophage infiltration increased and exosome (EXO) secretion increased in the lipopolysaccharide (LPS)-induced acute kidney injury (AKI) model. **a**, **b** Creatinine and urea nitrogen levels in LPS-induced AKI model after 12 and 16 h, respectively. **c–e** Neutrophil gelatinase-associated lipocalin (NGAL) and vascular cell adhesion molecule-1 (VCAM-1) expression in renal cortex by western blotting in LPS -induced AKI model. **f** Expression of macrophage marker, F4/80, was detected by immunohistochemistry in LPS-induced AKI. Scale bar ×100 and ×400. **g** CD63 expression in renal cortex EXOs of LPS-induced AKI model was detected by western blot. **i** CD63 was up-regulated in LPS-induced AKI. Scale bar ×400. Data represent the mean ± SEM for groups of 8 mice or independent experiments. ^*^P < 0.05, ^**^P < 0.01 versus Con

The LPS-AKI model was established in C57BL/6 male mice by intraperitoneal injection of LPS. Creatinine and urea nitrogen levels of the mice in the LPS treatment group increased significantly compared with the control group. In addition, in the LPS treatment group, the renal injury marker, NGAL, and endothelial cell injury marker, VCAM-1, were up-regulated at the protein levels. These results fully verify that the LPS-AKI model was successfully established in mice (Fig. 2a, e). Additionally, immunohistochemistry revealed that the expression of the macrophage marker, F4/80, increased in the LPS-treated group, indicating macrophage infiltration increased in LPS-AKI (Fig. 2f). Western blotting and immunohistochemistry showed that the expression of the EXO marker, CD63, was up-regulated in renal cortex and glomeruli, respectively, indicating that the secretion of EXOs in the LPS-AKI model was increased (Fig. 2g, i). In addition, partial colocalization of CD68 and CD63 was demonstrated by staining in successive kidney sections, further confirming that some of the exosomes produced in LPS-AKI are derived from macrophages (Additional file 1).

### EXOs from LPS-stimulated macrophages can induce the damage of glomerular endothelial cell in vitro

**Fig. 3:**
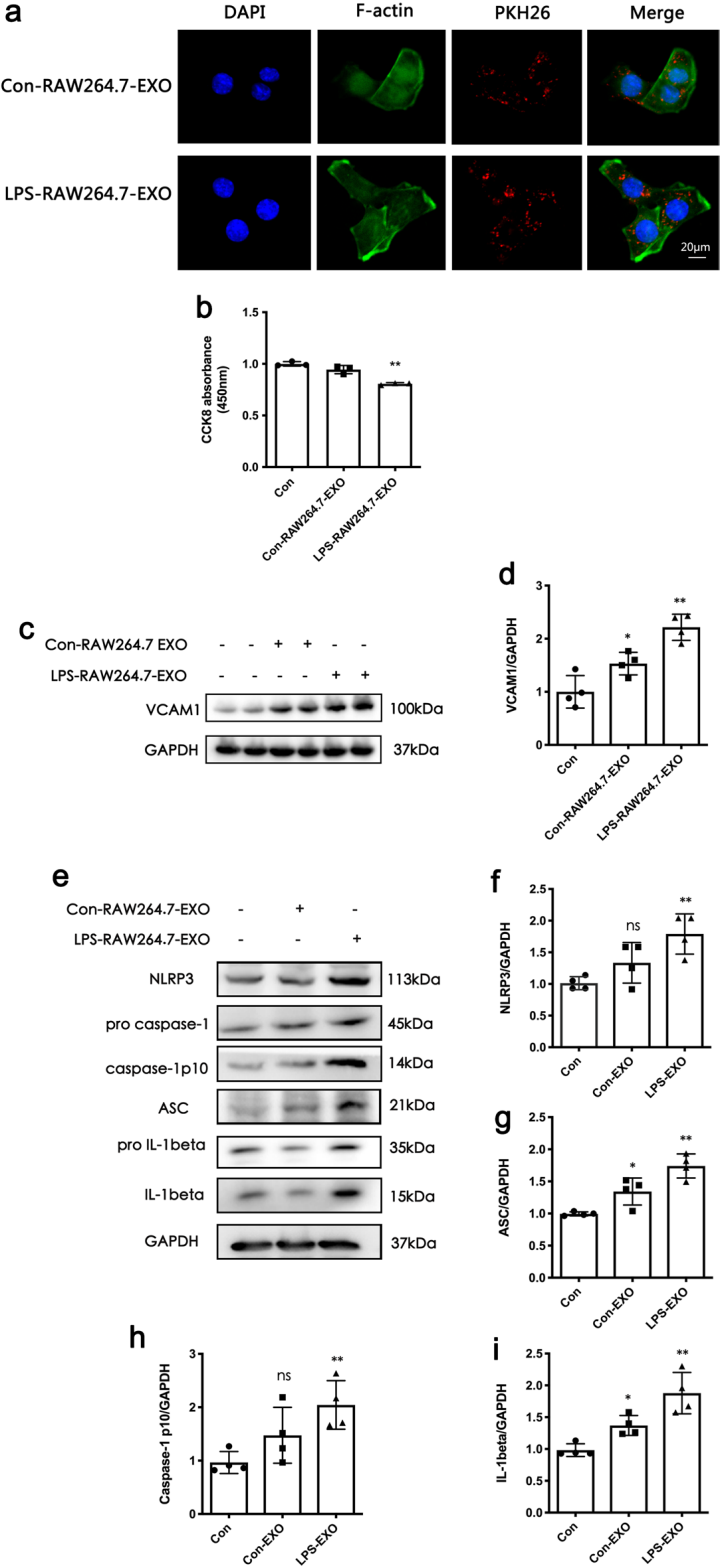
EXOs from lipopolysaccharide (LPS)-stimulated RAW264.7 can induce the damage of glomerular endothelial cell. **a** Confocal image of glomerular endothelial cells with 5 µg/mL PKH26 labeled RAW264.7-EXO for 24 h. Rat glomerular endothelial cells (RGECs) under a confocal microscope can internalize EXOs derived from macrophages. **b** Cell viability of RGECs as detected by cell counting kit 8. **c **and** d** Western blotting analysis of vascular cell adhesion molecule-1 (VCAM-1). **e–h** The expression of nucleotide-binding oligomerization domain-like receptor family pyrin domain containing 3 (NLRP3) inflammasomes and interleukin (IL)-1β in endothelial cells was detected by western blotting stimulated by EXOs produced by RAW264.7. Data represent the mean ± SEM in independent experiments. ^*^P < 0.05, ^**^P < 0.01 versus Con

Observation under a confocal microscope showed that EXOs from RAW264.7 can be internalized by RGECs in vitro (Fig. 3a). Moreover, EXOs from LPS-stimulated RAW264.7 can cause a decrease in cell viability in RGECs and the upregulation of VCAM-1 expression (Fig. 3b, d). In addition, we also found that EXOs from LPS-stimulated RAW264.7 induced the activation of NLRP3 inflammasomes in RGECs, including the upregulation of NLRP3, ASC, Caspase-1, and IL-1β (Fig. 3e–h).

### EXOs produced by LPS-stimulated macrophages can cause kidney injury in vivo

**Fig. 4:**
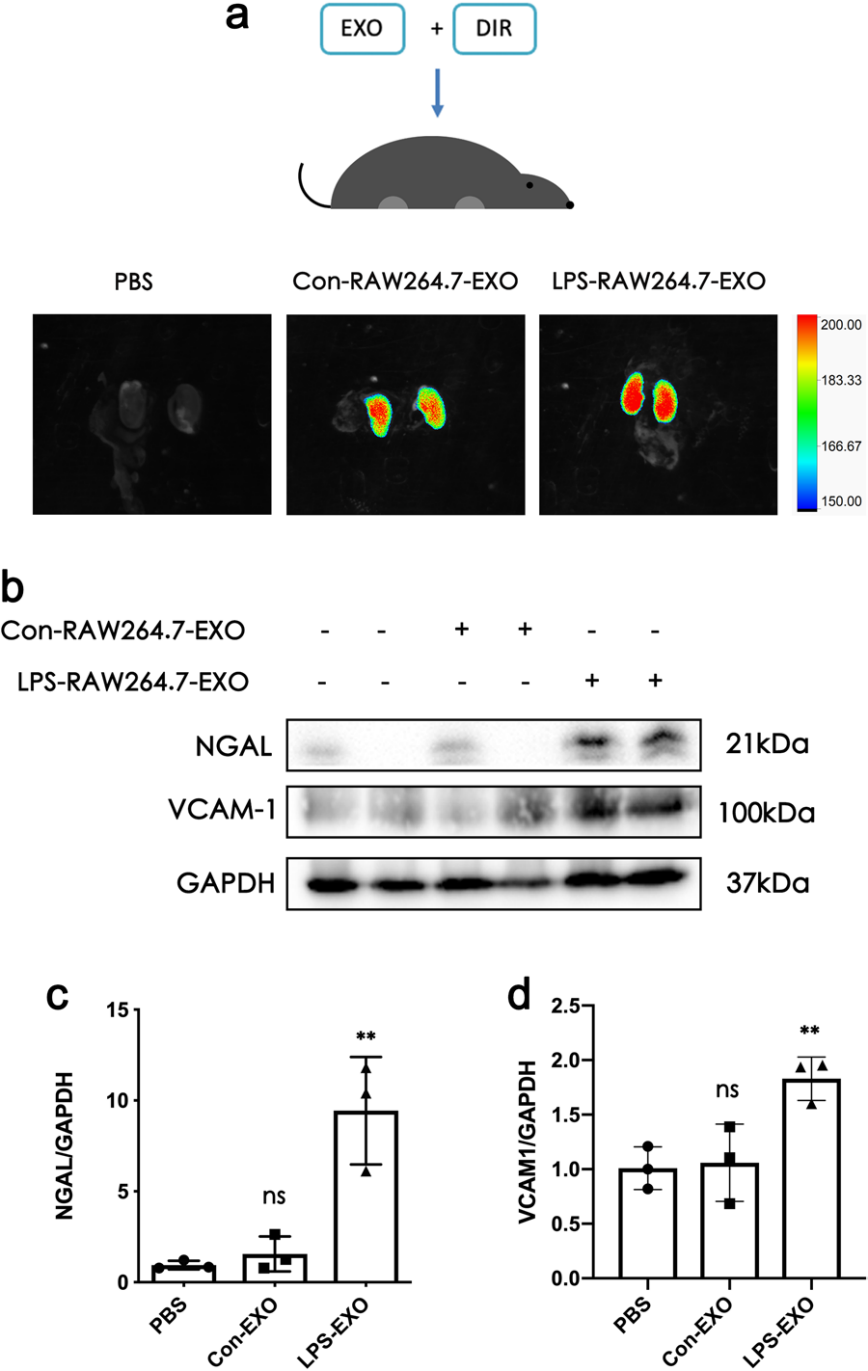
EXOs from lipopolysaccharide (LPS)-stimulated RAW264.7 can cause kidney damage in vivo. **a** Trace analysis of the metabolism (in vivo) of DIR-labeled EXOs injected into C57BL/6 mice via the tail vein. **b–d** Western blot analysis of neutrophil gelatinase-associated lipocalin (NGAL) and vascular cell adhesion molecule-1 (VCAM-1) after RAW264.7 exosomes extraction and injection into mice. Data represent the mean ± SEM in independent experiments. ^*^P < 0.05, ^**^P < 0.01versus Con

EXOs were injected mice through the tail vein and then entered the kidneys (Fig. 4a). After EXOs from LPS-stimulated RAW264.7 was injected into mice, the expression of NGAL and VCAM-1 was significantly upregulated in renal cortex (Fig. 4b, d). This indicated that EXOs from LPS-stimulated RAW264.7 can also cause kidney damage in vivo.

### ASM inhibition can reduce the production of macrophage EXOs and the damage of endothelial cells caused by LPS-stimulated macrophage EXOs

**Fig. 5:**
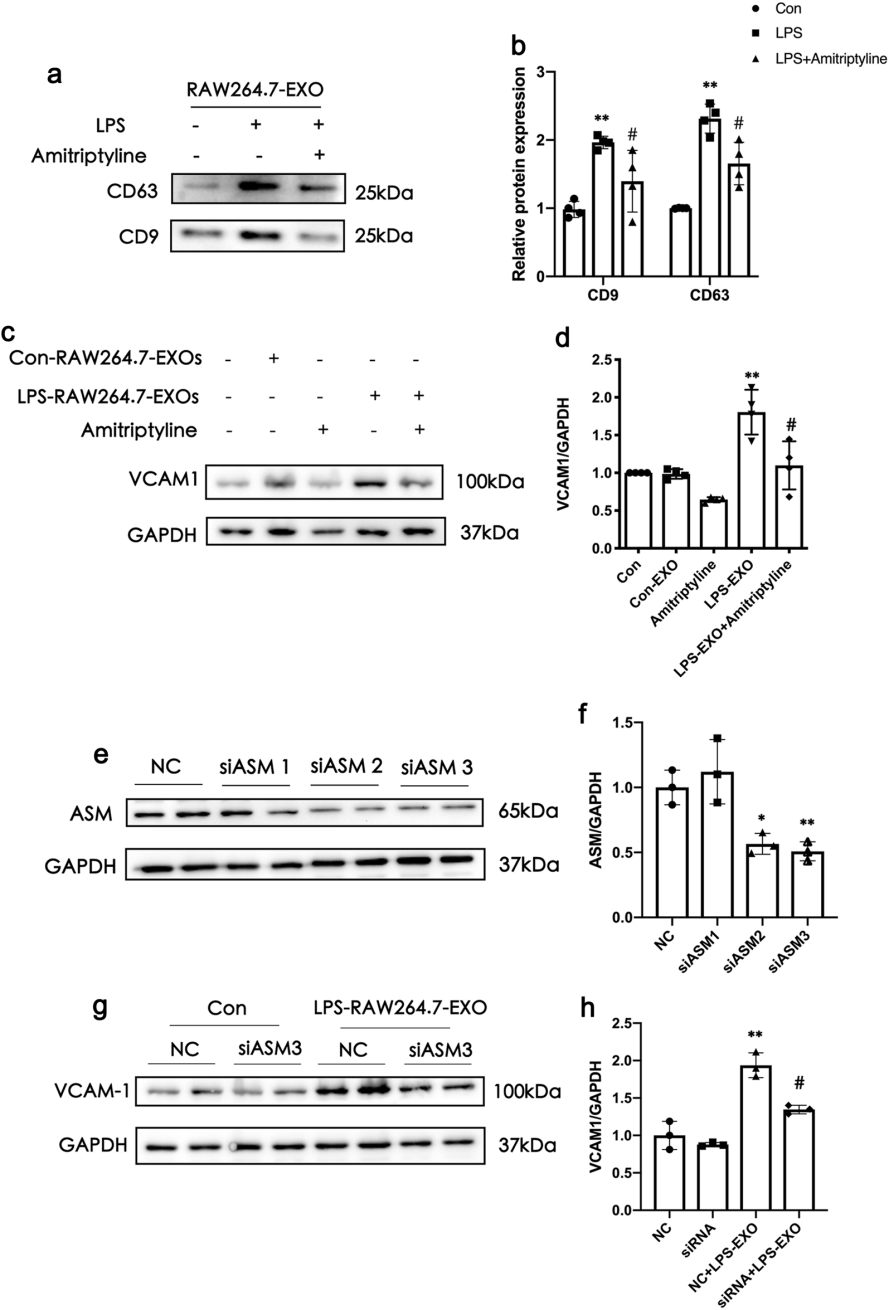
Acid sphingomyelinase (ASM) inhibition can reduce the production of macrophage exosomes and reduce the damage of endothelial cells caused by lipopolysaccharide (LPS)-stimulated macrophage exosomes. **a**, **b** Western blot analysis of the expression of exosome markers, CD9 and CD63. N = 3, ^**^P < 0.01versus Con, ^*#*^*P* < 0.05versus LPS group. Data are mean ± SEM. **c **and** d** Expression of endothelial cell damage index marker, vascular cell adhesion molecule-1 (VCAM-1). **e** and **f** ASM knockdown by small interfering ribonucleic acid in rat glomerular endothelial cells (RGECs). **g **and **h** Expression of VCAM-1 in endothelial cells after ASM knockdown in RGECs and LPS-RAW264.7-EXO stimulation. Data represent the mean ± SEM in independent experiments. ^**^P < 0.01versus NC group in Con, ^*#*^*P* < 0.05versus the NC group in LPS-RAW264.7-EXO

After RAW264.7 cells were pretreated with the ASM inhibitor, amitriptyline, and then stimulated with LPS, they produced fewer EXOs than those stimulated with LPS alone (Fig. 5a, b). In addition, we studied the effect of ASM on endothelial cell by pretreating RGECs with amitriptyline and then used EXOs from LPS-stimulated RAW264.7 to stimulate endothelial cells and found that endothelial cell damage was reduced. Furthermore, knocking down the expression of ASM on endothelial cells, the endothelial cell damage was reduced, indicating that ASM not only participates in the regulation of macrophage EXOs secretion, but also further affects the interaction of macrophage EXOs and endothelial cells.

### ASM deletion protects glomerular endothelial cell injury in LPS-AKI model

**Fig. 6:**
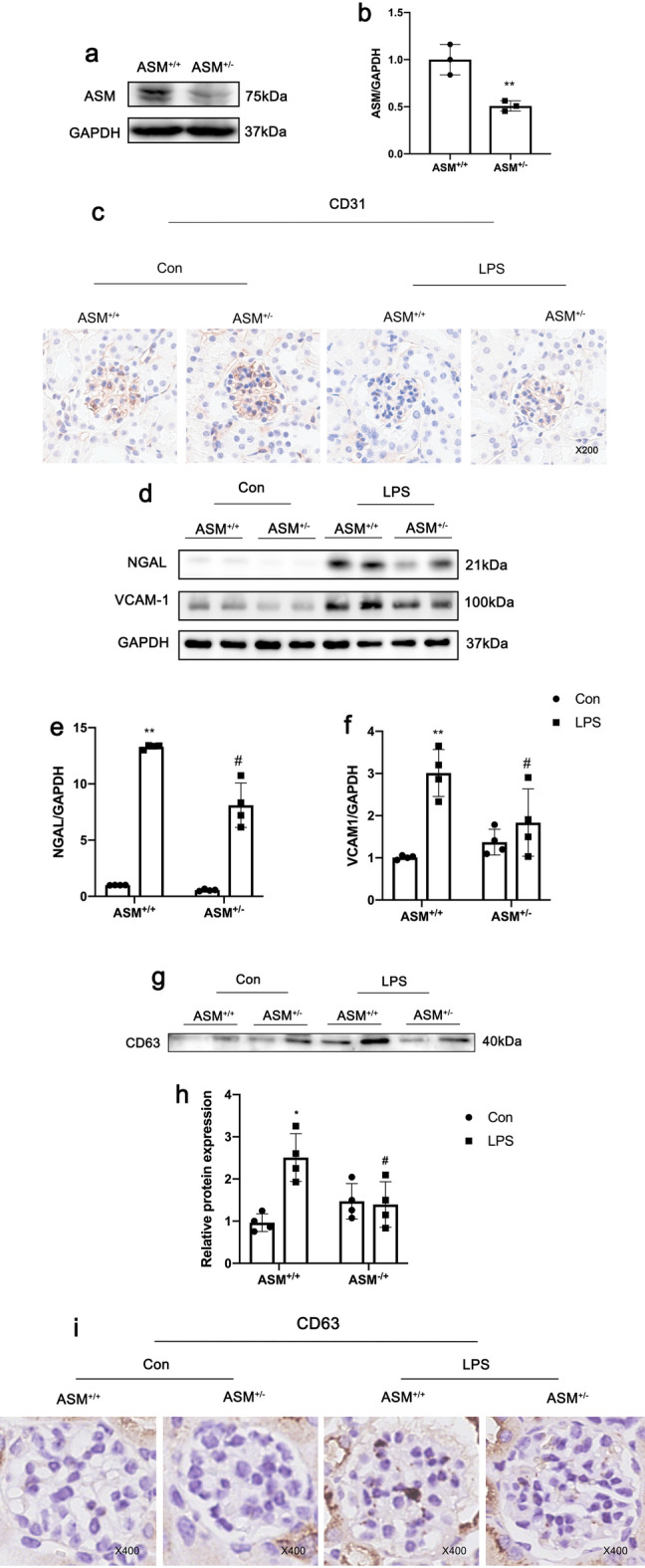
ASM deletion protects glomerular endothelial cell injury in LPS-AKI model. **a** and **b** Expression of acid sphingomyelinase (ASM) in renal cortex of ASM^+/−^ mice was detected by western blotting. c. CD31 immunohistochemical analysis in renal tissues. **d-f** Expression of neutrophil gelatinase-associated lipocalin (NGAL) and vascular cell adhesion molecule-1 (VCAM-1) in ASM^+/−^ mice and lipopolysaccharide (LPS)-acute kidney injury (AKI). **g–i** Western blot analysis of CD63 in kidney exosomes in the LPS-AKI model and CD63 immunohistochemical analysis in renal tissues. Data represent the mean ± SEM in independent experiments. ^*^P < 0.05, ^**^P < 0.01 versus Con or LPS group

We have confirmed that ASM can regulate the secretion of macrophages in vitro, but it is unclear whether the specific regulation of ASM affects LPS-AKI. Subsequently, ASM^+/−^ mice were used to further verify this hypothesis. Partial knockout of ASM in the kidney was detected by western blotting of proteins from ASM^+/−^ mice (Fig. 6a). In the LPS groups, immunohistochemistry indicated CD31 in ASM^+/+^ mice was reduced compared with ASM^+/−^ mice and western blotting indicated both NGAL and VCAM-1 showed a downward trend in ASM^+/−^ mice compared with ASM^+/+^ mice (Fig. 6c, f). This shows that partial knockout of ASM has a protective effect on the LPS-AKI model. Furthermore, in LPS-treated ASM^+/−^ mice, renal cortex EXOs were lower than those in ASM^+/+^ group (Fig. 6g, h). Immunohistochemistry also indicated CD63 infiltration in ASM^+/−^glomeruli was reduced compared with ASM^+/+^ mice (Fig. 6i).

## Discussion

The main mechanisms of sepsis-associated AKI are local blood insufficiency and tissue inflammation [16]. The cytokine storm during sepsis leads to the injury of multiple organs, including the kidney [17]. Macrophages play an important role in AKI inflammation; however, the specific mechanism has not been fully explained [18]. This study investigated the role of EXOs secreted by macrophages in the injury of endothelial cells in sepsis-associated AKI, both in vitro and in vivo.

Macrophages are innate immune cells that play an important role in the occurrence and development of AKI [19, 20]. In the early stage of AKI, macrophages aggravate renal injury by promoting the aggregation of inflammatory cells and the expression of inflammatory mediators; in contrast, macrophages can also damage surrounding resident cells, such as endothelial cells through the transmission of information media [21, 22]. In the sepsis-associated AKI mouse model, the infiltration of macrophages and the secretion of EXOs in glomeruli increased. In vitro, stimulation of cultured macrophages with LPS also resulted in a significant increase in EXO secretion. These results suggest that the EXOs secreted by macrophages may be involved in the occurrence and development of sepsis-associated AKI.

EXOs contain nucleic acids, proteins, lipids, etc. [23]. These components are characteristics of the host cell and affect the function or state of the recipient cell after being internalized [24, 25]. Internalization is an important step for EXOs to participate in cell-to-cell communication and provides an opportunity for EXOs with special contents to exert their pathophysiological effects [26, 27]. In our study, EXOs produced by macrophages were not only internalized by endothelial cells, but also distributed in the kidney after injection into the mouse tail vein.

EXOs secreted by LPS-stimulated macrophages were extracted in vitro and co-incubated with endothelial cells. The results showed that EXOs could be internalized by endothelial cells and lead to endothelial cell dysfunction, including decreased cell viability, increased VCAM-1 expression, and NLRP3 inflammasomes activation. In animal experiments, EXOs from LPS-stimulated RAW264.7 were injected into mice through the caudal vein. Western blotting showed that the VCAM-1 expression increased in renal cortex, which further verified the conclusion of the in vitro experiment. In sepsis-associated AKI, infiltrating macrophages in glomeruli can secrete many EXOs which act on surrounding endothelial cells, resulting in endothelial cell damage. Of note, endothelial cell injury is related to NLPR3 inflammasomes activation-mediated pyroptosis.

Recent studies have found that ASM may be involved in the formation of early endosomes, and the ceramide produced by ASM hydrolyzing sphingomyelin is related to the regulation of EXO secretion [28, 29]. The inhibition of central sphingomyelinase and secretion of EXOs decreased. It is unclear whether ASM also plays a role in the formation of macrophage EXOs. Inhibition of macrophage ASM reduces the release of EXOs following LPS stimulation. This result also indicates that ASM is involved in the regulation of macrophage EXO secretion. In addition, by inhibiting the ASM of endothelial cells, the damage of LPS-RAW264.7-EXO to endothelial cells was reduced. This shows that ASM not only participates in the regulation of macrophage EXO secretion, but also plays a role in the effect of EXOs on recipient cells. The role of ASM in recipient cells may affect the internalization of EXOs or their internalization to the lysosome; however, the specific mechanism involved needs to be studied further. EXO secretion is reduced in ASM^+/−^ mice. In addition, partial knockout of ASM has a protective effect against sepsis-associated AKI, which further confirms the conclusions of our in vitro experiments.

**Fig. 7:**
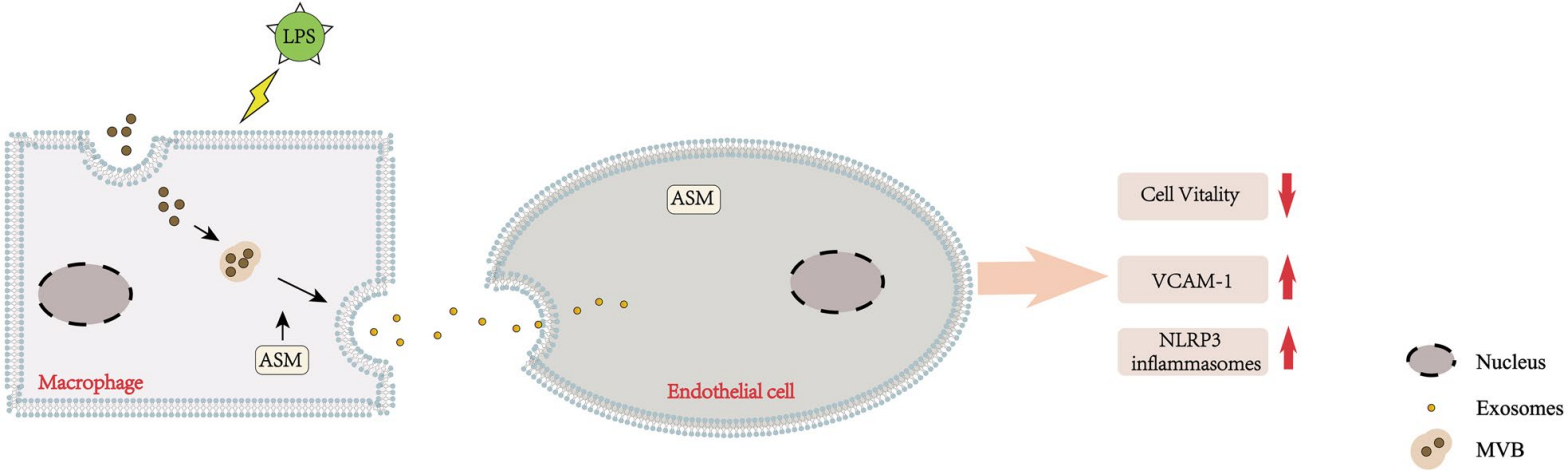
Schematic diagram showing how macrophage-derived exosomes affect endothelial cell function in sepsis-associated acute kidney injury

In conclusion, this study confirms that in sepsis-associated AKI, glomerular infiltrating macrophages can release many harmful EXOs, which are internalized by surrounding endothelial cells, resulting in the endothelial cell injury (Fig. 7). ASM was involved in regulating the secretion of EXOs by macrophages and their internalization by endothelial cells and may be a therapeutic target in sepsis-associated AKI.

## Materials and methods

### Animal experiment

All experimental protocols on animals were approved by the Ethics Committee of Tongji Medical College of Huazhong University of Science and Technology (Approval No. 2663). Experiments were carried out with 6-8-week-old male C57BL/6 mice (SPF Biotechnology, Beijing, China). All animals were cultivated in a specific pathogen-free environmental animal room at Tongji Medical College, Huazhong University of Science and Technology. All surgical operations were performed after anesthesia was induced using 1% pentobarbital.

For LPS-AKI model, the mice were divided into three groups (n = 8 per group): phosphate-buffered saline (PBS) group, LPS-12 h group (LPS 20 mg/kg for 12 h), and LPS-16 h group (20 mg/kg for16 h). The mice were anesthetized, and blood was collected. Creatinine and urea nitrogen levels were measured in the blood, and the kidney tissue was collected for further analysis.

For experiment in ASM knock out mice, the mice were divided into four groups (n = 6 per group): PBS group in ASM^+/+^ mice, PBS group in ASM^+/−^ mice, LPS (20 mg/kg for16 h) in ASM^+/+^ mice, and LPS (20 mg/kg for16 h) in ASM^+/−^ mice. The mice were anesthetized, and blood was collected. Creatinine and urea nitrogen levels were measured in the blood, and the kidney tissue was collected for further analysis.

For in vivo experiments on EXOs injection, the mice were divided into three groups (n = 3 per group): PBS group, control-RAW264.7-EXO injection group (40 µg) and LPS-RAW264.7-EXO injection group (40 µg). EXOs were injected into mice through tail vein. After 24 h, blood was collected under anesthesia to detect creatinine and urea nitrogen, and kidney tissue was collected for subsequent analysis.

### Cell culture

Rat glomerular endothelial cells (RGECs) were a gift from Shandong University; they were cultured in Dulbecco’s Modified Eagle Medium F-12 (DMEM-F12) with 1% penicillin streptomycin (Gibco, Carlsbad, CA, USA) and 10% fetal bovine serum (FBS; Science Cell, USA). RAW264. 7 cells were purchased from American Type Culture Collection and culture in DMEM medium with 1% penicillin streptomycin (Gibco) and 10% FBS (Science cell).

For the use of the ASM inhibitor amitriptyline, pretreatment with amitriptyline (20 µM) 0.5 h followed by other stimuli (LPS, or EXOs). The RGECs was knocked down ASM with siRNA (sequence: GCTACCGTTTACCAAAT) and then stimulated with EXO. The siRNA was transfected into RGECs with Lippo 2000 (thermo, USA), and the solution was changed 4 h after transfection.

### Isolation and characterization of EXOs

RAW264.7 cells were cultured in DMEM containing EXO-free FBS (ultracentrifugation at 120,000 ×*g* for 12 h) before LPS stimulation. RAW264.7 cells were cultured to 80% confluence, and then the control group was not given any treatment. The LPS group was stimulated with 1 µg/mL LPS (#L2880; Sigma, St Loui, MO, USA) for 24 h, and the supernatant was collected and subjected to a series of gradient centrifugation to extract the EXOs.

For the extraction of renal EXOs, 20 mg of renal cortex was digested with collagenase at 37 °C for 120 min. The samples were then subjected to EXOs extraction. The gradient centrifugation included 300 ×*g* for 10 min and 2000 ×*g* for 10 min, followed by 10000×*g* for 30 min. The supernatants were then centrifuged at 120000 ×*g* for 70 min. The pellets were washed once with PBS, centrifuged again at 120000×*g* for 70 min and resuspended in PBS (Type 70 Ti rotor; Beckman Coulter Optima, USA) .

The morphology of RAW264.7-EXO was examined using a transmission electron microscope (Hitachi HT770, Tokyo, Japan). Nanoparticle tracking analysis (NTA) was performed by Zetaview, PMX 110 (Particle Metrix, Meerbusch, Germany), and the protein levels was quantified using the BCA Protein Assay Kit (Aspen, Wuhan, China) following the manufacturer’s instructions.

### Macrophage-derived EXO labeling and uptake assay

RAW264.7-EXOs were incubated with PKH26 red fluorescent dye (Sigma) for 3 min, and the reaction was stopped with Dilute C. Subsequently, centrifugation was performed to obtain PKH26-labeledEXOs, and the cells were seeded in a 35 mm confocal culture dish and treated with 5 µg/mL PKH26-labeled EXOs. After culturing for 12 h, the cells were washed three times with PBS and fixed with 4% paraformaldehyde for 20 min. The cytoskeleton with phalloidin (Sigma), and the nuclei with 4′,6-diamidino-2-phenylindole (Aspen Biotech, Shanghai, China) for 10 min. The internalization of EXOs was observed under a confocal microscope.

RAW264.7-EXO were purified and stained with DIR. After dyeing for approximately 30 min, the dye was stopped with PBS, and the excess dye was removed by centrifugation at 120000 ×*g* for 70 min. DIR-labeled EXOs were injected into C57BL/6 mice via the tail vein. After 24 h, the distribution locations of DIR-labeled-EXOs in mice were observed using an in vivo fluorescence imaging system. After the preliminary observation, the mice were anesthetized, dissected and the kidneys were removed and observed directly using the in vivo fluorescence imaging system.

### Cell proliferation

Cell Counting Kit-8 (CCK8; Boster, China) was used to determine cell viability. RGECs were planted in 96-well plates according to the instructions. RAW264.7-EXO (5 µg/mL) were used to stimulate RGECs. After 24 h, the medium was changed and 10 μL CCK-8 mixed solution was added. After 1 h, the absorbance was measured at a wavelength of 450 nm.

### Western blotting analysis

Cells, kidney tissues, and purified EXOs were lysed in radioimmunoprecipitation assay lysis buffer containing protease inhibitors, and protein concentration was determined by BCA assay (aspen). The protein was subjected to sodium dodecyl sulfate polyacrylamide gel electrophoresis (Servicebio, China) and transferred to PVDF membrane (Millipore, USA). The membrane was blocked with 5% BSA (Biosharp, Hefei, China) for 1 h, and then the primary antibody was applied and incubated overnight at 4 ℃. The other reagents are listed here: anti- cluster of differentiation (CD) 9, anti-CD63, anti- interleukin (IL)-1β, anti- nucleotide-binding oligomerization domain-like receptor family pyrin domain containing 3 (NLRP3), anti-adaptor apoptosis-associated speck-like protein containing a caspase-recruitment domain (ASC), anti- vascular cell adhesion molecule-1 (VCAM-1), anti-ASM, anti-neutrophil gelatinase-associated lipocalin (NGAL) antibodies, all purchased from Abcam (USA); anti-caspase-1 (Santa Cruz Biotechnology, Santa Cruz, CA, USA); anti-GAPDH (Proteintech, Wuhan, China). Anti-mouse secondary antibody or anti-rabbit secondary antibodies (Proteintech) were used for detection in the BioSpectrum 600 imaging system (UVP, CA, USA).

### Histological analysis

After harvesting kidneys from male C57 BL/6 mice, they were fixed with 4% paraformaldehyde, dehydrated using a series of gradient ethanol, embedded in paraffin, and cut into 6 μm thick longitudinal sections. Sections were stained with hematoxylin and eosin, and histopathological changes were observed under a microscope.

### Statistical analysis

All data are expressed as mean ± SEM. The *t*-test was used to compare the differences between the two groups, and one-way or two-way analysis of variance was used for more than two groups. Statistical significance was set at *P* < 0.05. Statistical analysis was performed using GraphPad Prism 8.0 software (version 8.0; GraphPad Software, Inc., San Diego, CA, USA).

## Supplementary Information

**Additional file 1:** **Figure S1.** Successive sections stained with CD68 and CD63, respectively, showed that some CD63 could appear in the same location as CD68 in LPS-induced acute kidney injury.

## Data Availability

The datasets generated and analyzed during the current study are available from the corresponding author on reasonable request.
