# Supplementary material for: Macrophage-derived exosomes mediate glomerular endothelial cell dysfunction in sepsis-associated acute kidney injury
Source: Cell Biosci. 2023 Mar 7;13:46. doi: 10.1186/s13578-023-00990-z (PMC9990300; doi:10.1186/s13578-023-00990-z)
Supplement: Supplementary file 1 — Additional file 1: Figure S1. Successive sections stained with CD68 and CD63, respectively, showed that some CD63 could appear in the same location as CD68 in LPS-induced acute kidney injury. [file 13578_2023_990_MOESM1_ESM.docx]

**Additional file figure**

**Figure S1**

**
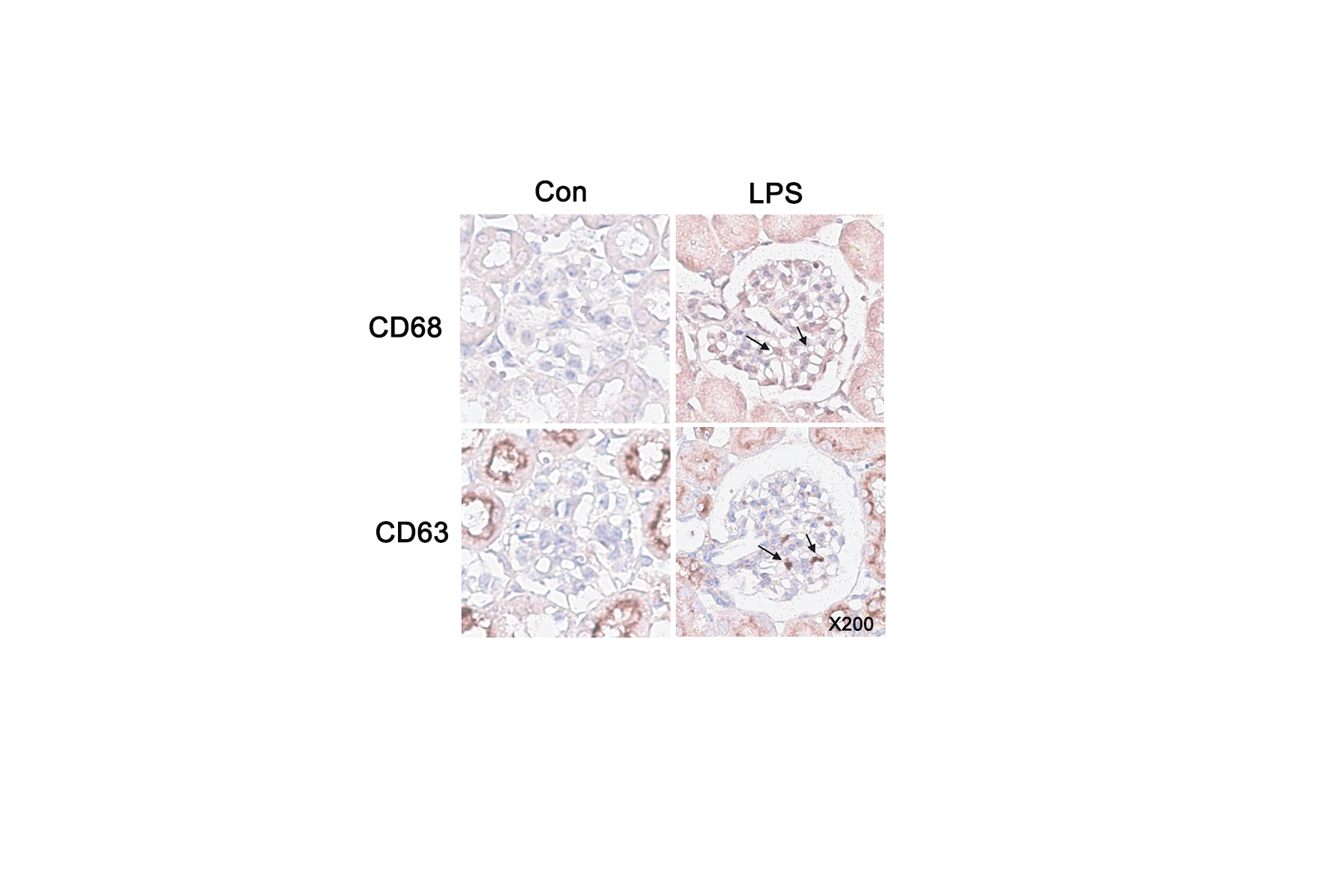
**

Successive sections stained with CD68 and CD63, respectively, showed that some CD63 could appear in the same location as CD68 in LPS-induced acute kidney injury.
